# *Trans* Fatty Acids Content in Breast Milk as a Marker of Their Short-Term Intake Within the Breastfeeding Mother’s Diet: A Single-Participant Pilot Study

**DOI:** 10.3390/nu18132177

**Published:** 2026-07-04

**Authors:** Edyta Jasińska-Melon, Hanna Mojska, Agnieszka Bzikowska-Jura

**Affiliations:** 1 Department of Nutrition and Nutritive Value of Food, National Institute of Public Health NIH—National Research Institute, Chocimska Str. 24, 00-791 Warsaw, Poland; hmojska@pzh.gov.pl; 2 Department of Clinical Dietetics, Faculty of Health Sciences, Medical University of Warsaw, Ciolka Str. 27, 01-445 Warsaw, Poland

**Keywords:** *trans* fatty acids, breast milk, GC-MS, a 10-day reconstructed daily diet, correlations, breastfeeding twins

## Abstract

**Introduction:** Breast milk is the best food for a growing infant during the first 6 months of life. The presence of *trans* fatty acids (TFAs) in breast milk can interfere with the synthesis of long-chain polyunsaturated fatty acids (LC-PUFAs), increasing the risk of developing, among other issues, asthma or atopic dermatitis. TFAs are not synthesised *de novo* in the human body. Their content in breast milk may be a good marker of short-term dietary intake of these compounds by breastfeeding mothers. However, the literature shows differences in the assessment of the relationship between dietary TFAs intake and TFAs content in breast milk. Furthermore, the decrease in the TFAs content in food observed recently seems to make it impossible to use Craig-Schmidt’s formula to estimate the TFAs content in the diet or in breast milk. The aim of this study was to confirm the possibility of using TFAs content in breast milk as a marker of their short-term intake within the breastfeeding mother’s diet, together with an attempt at preliminary quantitative determination of the relationship between these parameters. **Materials:** The study material was collected from a single breastfeeding mother and included 10 breast milk samples and 10 samples of daily food rations reconstructed based on the 24 h food consumption survey. **Methods:** The content of fatty acids, including TFAs, was determined by gas chromatography–mass spectrometry (GC-MS). **Results:** The TFAs content in the whole-day mother’s diet and in 100 mL of breast milk ranged from 0.11 to 0.54 g/day and from 0.02 to 0.07 g, respectively. A strong statistically significant (*p* < 0.05) positive correlation between these parameters was found. Equations for an exploratory linear relationship between the TFAs content in a breastfeeding mother’s diet and the concentration of these fatty acids in breast milk have been developed. Due to the small number of samples, these data should be interpreted very cautiously and validated in a larger cohort. **Conclusions:** This single-participant pilot study suggests that TFAs content in breast milk may be a marker of the dietary intake of these compounds from the previous day. It seems that the dietary habits of breastfeeding mothers of twins are a significant factor influencing the composition of breast milk and, consequently, the nutritional quality of breastfed infants.

## 1. Introduction

The World Health Organization (WHO) recommends exclusive breastfeeding for the first six months of a child’s life and continued breastfeeding until the end of the second year of life or longer (as long as the mother and child both want) with the gradual inclusion of appropriate complementary food during the second half of the child’s first year [1]. Breastfeeding is the best way to feed newborns and infants. Breast milk contains all the necessary nutrients, fully meets the baby’s needs at each stage of development, and guarantees proper growth and development [2]. 

Breast milk composition is influenced by many factors, both physiological and lifestyle-related, including the age of breastfeeding women, number of deliveries, pregnancy duration, lactation period, feeding time during the day, infections and metabolic diseases, and the use of specific medicines. However, the most significant factors influencing the composition of breast milk are a woman’s diet and her nutritional status before, during, and after pregnancy. This especially relates to the fat fraction of breast milk. The fatty acids found in human breast milk come from three sources: *de novo* synthesis in breast cells, adipose tissue and the liver, and from the breastfeeding mother’s diet [3].

Breast milk may contain *trans* fatty acids (TFAs or *trans* fats), which are defined as *“fatty acids with at least one non-conjugated (namely interrupted by at least one methylene group) carbon-carbon double bond in the trans configuration”* [4]. Their presence in tissues and body fluids, including breast milk, depends exclusively on diet, because the human body does not have enzymatic systems able to create *trans* configuration bonds. Depending on their source, TFAs are classified as ruminant *trans* fatty acids, (r-TFAs) or industrially produced *trans* fatty acids (i-TFAs). The first ones are produced in the rumen of ruminants and can be found in the milk and meat of those animals. The main source of i-TFAs in the human diet comes from partially hydrogenated fats/oils [5].

TFAs are a known risk factor for cardiovascular diseases, including ischemic heart disease (IHD) [6,7]. Studies have shown a relationship between TFAs consumption and, among other issues, type 2 diabetes [8], overweight and obesity [9,10,11], metabolic dysfunction-associated steatotic liver disease [12], cancers [13,14], and neurodegenerative disorders [15,16]. The negative effect of TFAs on fertility in both men [17] and women [17,18] is also highlighted. For infants’ development, it is important to note that TFAs may inhibit Δ-6 desaturase enzyme activity, thereby disrupting the synthesis of omega-3 and omega-6 long-chain polyunsaturated fatty acids (LC-PUFAs), including docosahexaenoic acid (DHA, C22:6 *n*-3) and arachidonic acid (ARA, C20:4 *n*-6), from their precursors α-linolenic acid and linoleic acid (LA, C18:2 *n*-6), respectively. The disruption of DHA synthesis leads to low tissue levels of this fatty acid. This, in turn, may disrupt development of the nervous system and the eye retina. Through inhibition of LC-PUFAs synthesis, TFAs may also shorten the duration of pregnancy and determine low birth weight in newborns [19]. Furthermore, in infants and small children they may increase the risk of developing, e.g., asthma or atopic dermatitis [19,20]. As early studies by Rosenthal and Doloresco [21] showed, the disruption of LC-PUFAs synthesis by TFAs is reflected in a reverse (negative) correlation between TFAs and LC-PUFAs levels. Until now, these relationships were demonstrated, among others, in blood lipid fractions in pregnant women [22], umbilical blood in neonates [23], and lipid fractions in plasma and erythrocyte membranes in the mother and foetus [24], as well as in the milk of breastfeeding mothers [25,26].

Depending on food sources and the amount of TFAs consumed by different population groups and in various regions of the world, a different TFAs content is observed in breast milk. According to Bahreynian et al. [27], the highest estimated pooled mean TFAs content was found in the breast milk of women from North America (6.99 g/L), followed by South America (2.36 g/L), Europe (1.82 g/L), and Oceania (1.21 g/L), while the lowest levels were found in the milk of women from Asia (0.71 g/L) and Africa (0.61 g/L). In the milk of women from Europe, the TFAs content ranged from 0.19% of all fatty acids (% *wt*/*wt*) (mature milk, Greece) to 4.40% *wt*/*wt* (mature milk, Germany) [20]. In the milk of Polish women, the TFAs share amounts to about 2.5% *wt*/*wt* in mature milk and 1.4–1.8% *wt*/*wt* in colostrum [28,29]. In a recently published study, the TFAs content in the milk of Polish women was at a level of approx. 0.5% *wt*/*wt* for C18:1 *n*-6 *trans* + C18:1 *n*-9 *trans* and about 0.10% *wt*/*wt* for C16:1 *trans*-7 [30]. The main TFAs identified in breast milk include *trans* isomers of oleic acid (C18:1), followed by palmitoleic acid (C16:1), and small quantities of *trans* isomers of polyunsaturated acids [20]. 

*Trans* fatty acids consumed in the maternal diet may be transferred directly to breast milk or stored in adipose tissue, and then released and transported to milk (TFAs can be transferred through both the blood–milk barrier and the placental barrier) [20]. According to Francois et al. [31], fatty acids consumed in a breastfeeding mother’s diet are rapidly transported to her milk (within 10–24 h). The levels of some fatty acids in breast milk may stay higher for up to three days after a meal. For TFAs, changes in their content in breast milk are observed within 12 to 36 h after a meal [32]. Therefore, it seems that TFAs content in breast milk may be used as a marker for their short-term intake within the diet. This is directly associated with assessment of the breastfed infant’s exposure on the adverse effects of TFAs.

It should be noted that the quantity and type of fatty acids in breast milk are very important. Fatty acids accumulated in the feeding period through breast milk will be used by a developing body, e.g., as an energy reserve material, structural material of cell membranes, or component of plasma lipids. Therefore, assessment of the short-term impact of TFAs intake in the breastfeeding mother’s diet on the content of these compounds in breast milk, including a quantitative determination of the relationship between these parameters, appear to be of crucial importance. Especially that there are limited studies presenting the impact of real TFAs intake on their content in breast milk. In many studies assessing the correlation between a diet and breast milk, the TFAs content in a diet was estimated based on survey data/food frequency questionnaires, and data on the TFAs content in individual food products [25,29,33,34,35,36,37,38,39]. However, the studies cited above show inconsistencies in the assessment of how the TFAs intake in breastfeeding women’s diets affects the concentration of these fatty acids in breast milk. Furthermore, the estimation method used in those studies is time-consuming and requires current data on the TFAs content in food products in a given country. In other studies, the total dietary TFAs intake was not reported, and only groups of products for which the consumed quantity was significantly correlated with the TFAs level in breast milk were identified [40,41,42,43,44,45]. In yet other studies [46,47], the TFAs intake in the breastfeeding mother’s diet was estimated using the formula proposed by Craig-Schmidt et al. [48]: % *trans* C18:1 in milk = 1.49 + 0.42 × % *trans* C18:1 in a diet from the previous day. However, according to the authors, this formula can only be used when the *trans* C18:1 content in breast milk exceeds 1.49% *wt*/*wt*. It is worth noting here that, together with a significant reduction in the use of partially hydrogenated oils (PHOs) in food, a global trend of reducing the TFAs (primarily *trans* C18:1) content in food products is observed [49,50,51,52,53], which also results in a decrease in the total TFAs content in breast milk [20,46,54]. In consequence, it appears that today it is not possible to use the formula by Craig-Schmidt et al. to estimate the TFAs intake with a breastfeeding mother’s diet, based on the results of these fatty acids content in milk. It also appears that the use of the above formula is associated with the risk of under- or overestimating the TFAs content in breast milk. 

Therefore, we carried out a study aimed at assessing the correlation between the analytically determined TFAs content in human breast milk samples collected from a single mother over 10 consecutive days of breastfeeding and the analytically determined content of these fatty acids in the reconstructed daily food rations of the study participant from the previous days. The aim was to confirm the possibility of using TFAs content in breast milk to assess their short-term intake within the breastfeeding mother’s diet, together with an attempt at quantitative determination of the relationship between these parameters.

## 2. Materials and Methods

### 2.1. Study Design

The study involved a 36-year-old woman from the Mazovia Voivodeship, who delivered healthy twins via caesarean section at 38 weeks of pregnancy. Her pregnancy was physiologically normal, with no medical complication for the mother or the foetuses, and the delivery took place on the date specified by a physician providing prenatal care. The inclusion criteria for the study were no diagnosed diseases, including metabolic, cardiovascular, gastrointestinal, and cancer disorders, no chronic medications associated with those disorders, and written consent to participation in the study. The study was performed in the third month of exclusive breastfeeding. At the time of the study, the participant’s body weight was 54 kg, and her height was 168 cm. Her body mass index (BMI) of 19.13 kg/m^2^ was calculated by dividing her body weight by her squared height (kg/m^2^). The woman had a university degree and, after learning the study aim and plan, she agreed to participate by signing her written consent. 

**Figure 1:**
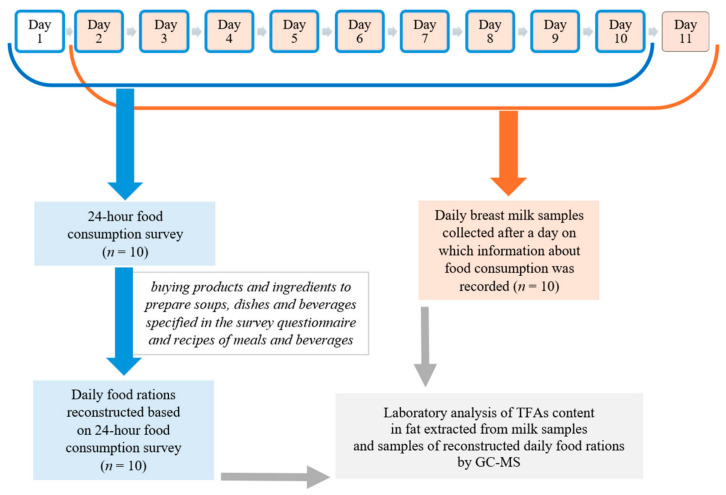
The research scheme. Explanations: the blue arrow shows reconstructed daily food rations samples collection; the orange arrow shows breast milk samples collection; the grey arrow shows the type of laboratory analysis.

The research scheme is shown in Figure 1.

All procedures performed in this study conformed to the ethical standard provided in the 1964 Helsinki declaration and its later amendments. The protocol was approved by the Bioethics Commission of the National Institute of Public Health NIH—National Research Institute (NIPH NIH—NRI) (Opinion No. 9/2021 from 30 June 2021). 

### 2.2. Food Consumption Records

On ten successive days of breastfeeding, the study participant completed a written food consumption survey questionnaire (24 h food consumption survey). She consumed her normal diet and she was not provided any recommendations or guidance on nutrition. She was thoroughly instructed how to complete the survey, including:

- For commercial products and beverages: characteristics of products and beverages consumed—their commercial name, name of the product brand and manufacturer, the weight and/or volume of the product’s commercial package, place of purchase, fat content (if applicable), e.g., 3.2% milk, 18% cream, etc., the size of the commercial product serving consumed in grams or millilitres, and a photograph of the label (if applicable).
- For soups: soup ingredients and the size of the soup serving consumed in grams.
- For dishes: dish ingredients and the size of the dish ingredient serving consumed in grams, e.g., boiled potatoes, rice, pork frikadelle, chicken breast.
- For beverages other than commercial beverages (beverages prepared at home, e.g., white coffee): characteristics of commercial products used to prepare that beverage—their commercial name, name of the product brand and manufacturer, the weight and/or volume of the product’s commercial package, fat content (if applicable), e.g., 3.2% milk, 18% coffee creamer, etc., the size of the commercial product serving used to prepare the beverages in grams or millilitres, and a photograph of the label (if applicable).

For soups, dishes and beverages other than commercial beverages (beverages prepared at home), the study participant delivered recipes of prepared meals and beverages, together with information on the list of ingredients used and their weight in grams or volume in millilitres, and for prepared dishes, additional information on the type or kind of cookware used to prepare the dish, the type of fat used to prepare the dish, and the cooking technique used, including cooking or frying time, baking/roasting temperature, etc.

The sizes of product servings consumed (including commercial products used to prepare beverages), soups and dish ingredients were weighed to a precision of 0.1 g using a Hoffen electric kitchen scale (model: KS-1137-B; serial number POJM210137; VERSHOLD POLAND Sp. z o.o., Warsaw, Poland). The volumes of commercial beverages (e.g., milk) used to prepare beverages were measured to a precision of 1 mL using a Pyrex kitchen measure made of borosilicate glass. The above data was recorded in the questionnaire of the 24 h food consumption survey.

### 2.3. Breast Milk Sample Collection

Before the start of the study, the participant received: instructions on breast milk sampling, polypropylene containers for breast milk, and a thermo-insulated container with a cooling insert. During ten successive days of breastfeeding, starting with the second day of collecting nutritional data (Figure 1), the study participant collected the whole-day milk samples (*n* = 10) over 10 consecutive days (Figure 1). Milk sampling started from the second day because fluctuations in TFAs levels in breast milk appear to occur 12 to 36 h after a meal. Milk samples were collected using a breast pump throughout the entire study day, each time after feeding the babies, from both breasts, and from different breastfeeding sessions. The children were breastfed on demand (responsive feeding)—there were no consistent feeding times throughout the day. Between feedings, the container with milk was stored in a fridge (4 °C). The minimum size of a milk sample from the entire day could not be smaller than 15 mL. When the proper amount of milk was collected (no less than 15 mL), the sample was stored frozen (−18 °C) away from light, and within 24 h was delivered to the laboratory of the National Institute of Public Health NIH—National Research Institute (NIPH NIH-NRI)—in conditions that protected the sample against thawing and without exposure to light, where they were stored in a low-temperature freezer (−80 °C) until analysis; however, they were stored for no longer than 1 month.

### 2.4. Reconstruction of Daily Food Rations

Based on the 24 h food consumption survey carried out over 10 successive days, the composition of successive daily food rations was reconstructed. The food rations were prepared within one month after the end of breast milk sample collection and the end of the collection of nutritional data. The products described in the 24 h food consumption survey and in the recipes of meals and beverages were bought in commercial retail stores in Warsaw. Soups and dishes were prepared using the cooking techniques described by the study participant in the questionnaire. Product, soup and dish serving sizes were weighed to a precision of 0.1 g on a technical scale AD 2000 Axis. Volumes of beverages were measured to a precision of 1 mL using a class A glass measuring cylinder Simax of 500 mL, manufactured in accordance with ISO 4788. Then, average laboratory samples were prepared from all products, meals and beverages consumed on one day. Preparation of the average laboratory samples included manual mixing of all ingredients and then, depending on need, the fragmentation, grinding and homogenisation of the entire material. Homogeneous average laboratory samples prepared this way, following another thorough (manual) mixing, were divided into subsamples for further analysis by weighing 100 g of material. The subsamples prepared this way were then frozen and stored at −20 °C until analysis; however, they were stored for no longer than two weeks. Before analyses, subsamples were thawed at room temperature and thoroughly (manually) mixed, and then the required amount of material from each subsample was weighed for analysis.

### 2.5. Determination of Nutrients and Fatty Acids Content, Including Trans Fatty Acids

#### 2.5.1. Determination of Nutrient Content in Reconstructed Daily Food Rations

The fat content in the samples of reconstructed daily food ratios was determined at the NIPH NIH-NRI laboratory by the extraction weighing method (Soxhlet method) using the extraction apparatus with the pre-hydrolysis attachment, as previously described [55]. The method was validated and accredited by the Polish Centre of Accreditation (accreditation certificate AB 509). The fat content was determined in two parallel determinations. The results were expressed in grams per 100 g of the reconstructed daily food ration (g/100 g). The quality criteria of the method were confirmed using the certified reference material of Canned Meat—150 g, FAPAS QC Material, Fera Science Ltd. T01122QC (Sand Hutton, England)—and satisfactory results of participation in proficiency tests (FAPAS). Protein content according to PB-39/LCH rev.3 of 30/09/2016, total ash content according to PB-49/LCH rev.2 of 08/11/2023, water according to PN-85/A-82100, and dietary fibre content according to AOAC 991.43:1994 were determined at the Silliker Polska Sp. z o.o. laboratory in Warsaw (accreditation certificate AB 462). The available carbohydrate content and the energy value of the reconstructed daily food ratios were determined as follows:

(1) The total carbohydrate content was calculated (g/100 g):

$$\begin{array}{l} Total\;carbohydrates\;(g/100\;g)=100-[Protein\;(g/100\;g)+Fat\;(g/100\;g)\;+\\ Total\;ash\;(g/100\;g)+Water\;(g/100\;g)] \end{array}$$

(2) The available carbohydrate content was calculated (g/100 g):

$$\begin{array}{l} Available\;carbohydrates\left(g/100\;g\right)=Total\;carbohydrates\left(g/100\;g\right)-\\ Total\;dietary\;fibre\;(TDF)\;(g/100\;g) \end{array}$$

(3) The energy value per 100 g of reconstructed daily food rations was calculated:

$$\begin{array}{c} Energy\;(kcal/100\;g\;of\;reconstructed\;daily\;food\;ration)=E\_fat\;(kcal/100\;g)\;+\\ E\_protein\;(kcal/100\;g)+\;E\_available\;carbohydrates\;(kcal/100\;g)+\;E\_fibre\;(kcal/100\;g)\; \end{array}$$

where

E_fat—energy from fat.
E_protein—energy from protein.
E_ available carbohydrates—energy from available carbohydrates.
E_fibre—energy from dietary fibre.

We assume that 1 g of protein provides 4 kcal of energy, 1 g of fat provides 9 kcal of energy, 1 g of available carbohydrates provides 4 kcal of energy, and 1 g of fibre provides 2 kcal of energy.

(4) The energy of reconstructed daily food rations was calculated:

$$\begin{array}{l} Energy\;(kcal/reconstructed\;daily\;food\;ration)=[Energy\;(kcal/100\;g)\;\times \\ Weight\;of\;whole\text{–}day\;diet\;(g)]/100 \end{array}$$

#### 2.5.2. Determination of Fatty Acids Content, Including TFAs in Reconstructed Daily Food Rations by Gas Chromatography–Mass Spectrometry (GC-MS) 

Fatty acids including TFAs were analysed as methyl esters (FAMEs) by gas chromatography using the Hewlett-Packard 7890A gas chromatograph (GC) (Agilent Technologies, Santa Clara, CA, USA) equipped with a capillary column CP Sil 88 (100 m long, 0.25 mm in diameter, film thickness of 0.20 μm; Agilent J & W GC Columns (Santa Clara, CA, USA) and the 5975C Inert mass spectrometer detector (MS), as described in a previous study [55]. In short, one microlitre (1 μL) of prepared fatty acids methyl ester extract in isooctane was injected into the column using a split injector (1:100 ratio) and helium as a carrier gas (20 mL/s; pressure of 43.4 psi). The oven temperature (ramp up at 5 °C/min, from 175 °C (hold for 40 min) to 220 °C (hold for 20 min) and the MS transfer line and injector temperature (250 °C) were preprogrammed. Identified peaks were verified by comparison against authentic standards (Supelco FAME Mix 37 Component; Sigma-Aldrich CHEMIE GmbH, St. Louis, MO, USA) and by mass spectrometry using Agilent ChemStation Software (version B.03.01). The method was validated and accredited by the Polish Centre of Accreditation (accreditation certificate AB 509). The quality criteria of the method were confirmed using the certified reference material BCR-162R (Soya-maize oil blend, 5.5 g; Sigma-Aldrich, St. Louis, MO, USA) and by satisfactory results of participation in proficiency tests (FAPAS). 

#### 2.5.3. Determination of Nutrient Content in Breast Milk

Human breast milk samples were thawed once at room temperature, protected from light, and then homogenised by thorough mixing. The twisted container was mixed manually by performing 10 sequences involving turning the container 180° and returning it to its starting position with its cap up. From samples prepared this way, 1 mL was taken for the fatty acids analysis in two parallel determinations, as described in Section 2.5.4. The remaining amount, after securing the container with parafilm at the point of the cap’s contact with the container, was transferred within a maximum of 2 h, in refrigerated conditions with no light access, to the Laboratory of Human Milk and Lactation Research at Warsaw Medical University’s Regional Human Milk Bank in the Holy Family Specialist Hospital in Warsaw, for macronutrient determination.

Determination of nutrients (fat, total protein, true protein, and carbohydrates), dry matter, and energy measurements in breast milk samples was conducted using the MIRIS human milk analyser (HMA MIRIS, Uppsala, Sweden) according to the manufacturer’s recommendations. The principle of HMA operation is based on mid-infrared (MIR) spectroscopy. This is a technique used to analyse the molecular composition of substances by measuring infrared light absorption within the MIR range, from 2500 to 25,000 nanometres. Prior to analysis, a daily calibration check was performed using the calibration solution (MIRIS check) provided by the supplier. Before analysis, each sample (*n* = 10) was heated to 40 °C and then homogenised for 1.5 s/1 mL of the probe using a sonicator (Milk Homogenizer, MIRIS, Uppsala, Sweden). The content of each component in each sample was determined three times. For analysis, 3 mL of milk was collected for each determination (9 mL in total). The homogeneous milk sample was injected into a flow cell and measured for 60 s. The concentrations of all macronutrients (fat, carbohydrates, protein—total and true) and dry matter content in each sample were expressed as the mean of three replicate measurements in grams per 100 mL of breast milk (g/100 mL). Energy was expressed as kilocalories (kcal/100 mL) and calculated at 9 kcal/g for fat, 4 kcal/g for protein, and 4 kcal/g for carbohydrates.

#### 2.5.4. Determination of Fatty Acids Content, Including TFAs, in Breast Milk by Gas Chromatography–Mass Spectrometry (GC-MS)

Fat from 1 mL of mixed milk sample was extracted by the Folch et al. method [56] with a mixture of chloroform and methanol (2:1) (Avantor Performance Materials S.A., Gliwice, Poland) containing 0.02% of butyl-hydroxytoluene (2,6-tert-butyl-4-methylphenol, BHT, ≥99.0%, GC, powder) (Sigma-Aldrich CHEMIE GmbH, St. Louis, MO, USA). 

Fatty acids were analysed as methyl esters (FAMEs). The methylation procedure was as follows: Organic extracts were evaporated at 40 °C under a gentle nitrogen stream, saponified with 0.5 mL of potassium hydroxide in methanol (0.5 N) (Avantor Performance Materials S.A., Gliwice, Poland) at 80 °C in an electric multi-block heater for 10 min, and subsequently methylated with 1 mL hydrochloric acid in methanol (3 N) (Sigma-Aldrich CHEMIE GmbH, St. Louis, MO, USA) at 85 ± 2 °C for 15 min. After cooling to room temperature, FAMEs were extracted with 1 mL of isooctane (2,2,4 trimethylopentane) (Avantor Performance Materials S.A., Gliwice, Poland) and vortexed for 10 s, before adding sodium chloride solution (40 g/100 mL in distilled water) (Avantor Performance Materials S.A., Gliwice, Poland) heated to a temperature of 50–60 °C. Then, 200 μL of the upper (FAMEs) fraction and 200 μL of isooctane were transferred into a 1.5-millilitre glass vial, and loaded into the gas chromatography autosampler.

Fatty acids, including TFAs, were analysed by gas chromatography using a Hewlett–Packard 7890A gas chromatograph (GC) (Agilent Technologies, Santa Clara, CA, USA) equipped with a capillary column CP Sil 88 (100 m long, 0.25 mm in diameter, film thickness of 0.20 μm; Agilent J & W GC Columns, Santa Clara, CA, USA) and 5975C Inert MS, as described in Section 2.5.2. with slight modification of the oven temperature program: from the initial temperature of 80 °C for 2 min, the temperature increased from 80 °C to 175 °C at 20 °C/min; the sample was kept at an isotherm of 175 °C for 40 min; the temperature increased to 220 °C at 5 °C/min; and it was maintained at an end temperature of 220 °C for 15 min (the total analysis time was 71 min). 

### 2.6. Statistical Analysis

The nutrient content and energy of the reconstructed daily food rations are presented in g/reconstructed daily food ration and in kcal/reconstructed daily food ration, respectively. For milk samples, the results of nutrient content and energy are presented in g/100 mL and in kcal/100 mL of milk, respectively. The fatty acids content, including TFAs, are presented as the percentage share of those fatty acids in the sum of all identified fatty acids (% *wt*/*wt*) and in g/reconstructed daily food ration. Additionally, TFAs are presented in g/100 mL of breast milk. 

Statistical analyses were conducted using Statistica software, ver. 13 (StatSoft Inc., Tulsa, OK, USA) from StatSoft Polska Sp. z o.o. with its registered office in Kraków (at Cystersów 9, 31-553 Kraków, Poland). Correlations between TFAs intake with diet and TFAs content in breast milk were estimated using Pearson’s r correlation coefficient and the Spearman correlation coefficient. Variable distributions were evaluated with a Shapiro–Wilk test. A p-value below 0.05 was adopted as statistically significant.

## 3. Results

### 3.1. Nutrient and Fatty Acids Content, Including TFAs, in Reconstructed Daily Food Rations 

**Figure 2:**
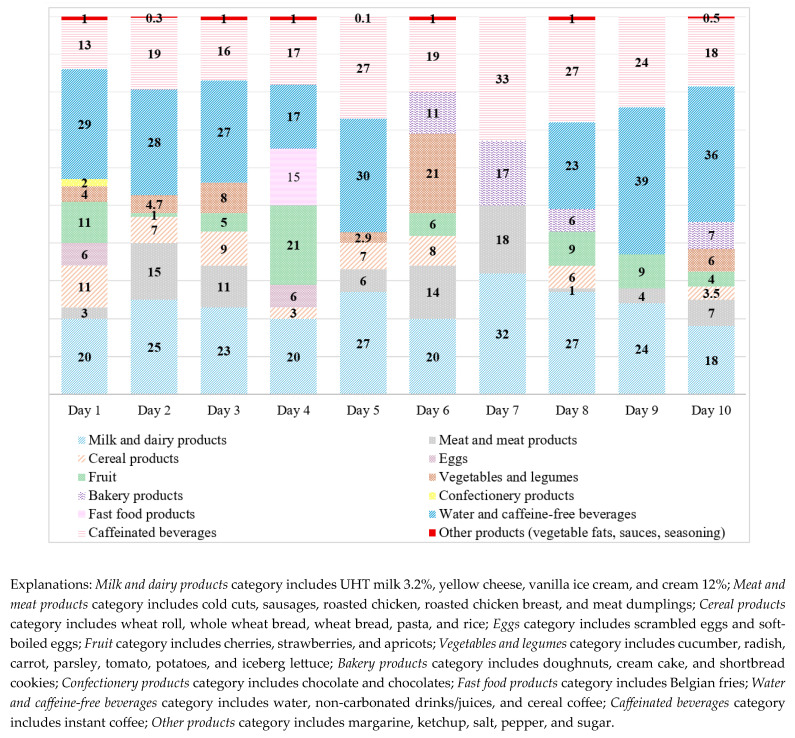
A percentage share (%) of individual food product groups in the daily breastfeeding mother’s diet.

Figure 2 presents a percentage share of individual food product groups in the breastfeeding mother’s diet.

Every day, the woman’s diet included food products from the milk and dairy products category. Products in the meat and meat products category were consumed with similar frequency (9 days). Consumption of cereal products (bread) and fruit was recorded on 8 of the 10 days. Consumption of vegetables and legumes was recorded on 6 days. Vegetable fats and seasoning were included rarely (5 and 3 days, respectively). Bakery products such as cakes and biscuits were consumed equally infrequently (4 days). Products from the confectionery category such as chocolates and fast food products like Belgian fries were consumed very rarely (1 day in each case). No fish or seafood was included in the diet of ten days. During the lactation period, the participant also did not consume salty snacks or drink carbonated drinks or alcohol, whereas she consumed caffeinated beverages (coffee) daily. A high proportion of fast food products was also recorded on the fourth day.

The weights, nutrient content, and energy of the reconstructed daily food rations are presented in Table 1.

The average (median, Me) weight of the study participants’ daily diet was 1664.4 g (including beverages) (range: 638.0–2355.0 g). The median energy value of the analysed daily food rations was 972 kcal (range: 198–1944 kcal/reconstructed daily food ration).

The median fat content was 35.8 g/reconstructed daily food ration (range: 5.1–79.2 g/reconstructed daily food ration). The percentage share of daily energy intake (%En) from that component ranged from 23% (Day 9) to 41% (Day 7). The percentage share of daily energy intake from other nutrients was, on average, 17%En (12–25%En) for protein, 46%En (41–53%En) for carbohydrates, and 2%En (1–4%En) for dietary fibre.

In the fat extracted from the reconstructed daily food rations, 36 fatty acids were identified. The content of selected fatty acids, including TFAs, in reconstructed daily food rations, as the percentage share of individual fatty acids in the sum of all identified fatty acids (% *wt*/*wt*), is presented in Table 2.

Significant differences in the content of both fatty acid groups and individual fatty acids were observed in the reconstructed daily food rations between individual days: saturated fatty acids (SFAs) 42.67–65.47% *wt*/*wt*, monounsaturated fatty acids (MUFAs) 29.15–43.89% *wt*/*wt*, polyunsaturated fatty acids (PUFAs) 3.25–14.65% *wt*/*wt*, and *trans* fatty acids (TFAs) 0.50–2.11% *wt*/*wt*. Among SFAs, myristic (C14:0), stearic (C18:0), and palmitic (C16:0) acids were present in the largest quantities. The percentage share of the daily energy intake from SFAs ranged from 15% to 22%En. Oleic acid (C18:1) predominated among MUFAs. Among PUFAs, linoleic acid (C18:2 *n*-6, LA) was present in the largest quantity. The determined LA content was several to several dozen times higher than that of α-linolenic acid (C18:3 *n*-3, ALA): 2.56–13.13% *wt*/*wt* vs. 0.24–1.92% *wt*/*wt*. LA consumption covered 3.5% of energy from food on average (range: 0.6–4.9%En). ALA consumption covered 0.3%En on average (range: 0.1–0.7%En). Long-chain polyunsaturated fatty acids *n*-3 (LC-PUFAs *n*-3) and *n*-6 (LC-PUFAs *n*-6) were present in all analysed samples of reconstructed daily food rations. Their content ranged between 0.29 and 1.99% *wt*/*wt* for the family of *n*-3 fatty acids, and 2.92–13.59% *wt*/*wt* for the family of *n*-6 fatty acids. In milligrams, the median eicosapentaenoic acid (C20:5 *n*-3, EPA) + docosahexaenoic acid (C22:6 *n*-3, DHA) intake in the diet was 4 mg/reconstructed daily food ration (range: 2–44 mg/reconstructed daily food ration). The median DHA intake was 2 mg/reconstructed daily food ration (range: 1–40 mg/reconstructed daily food ration). It was also observed that the *n*-6/*n*-3 fatty acids ratio ranged from approximately 6:1 to 25:1 and deviated from the beneficial ratio (approximately 2:1). 

**Figure 3:**
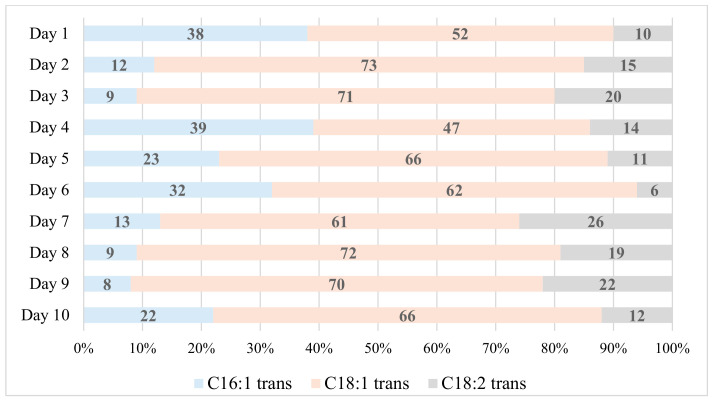
The percentage share of *trans* isomers of individual fatty acids in the pool of all identified *trans* isomers in samples of reconstructed daily food rations.

In all samples of reconstructed daily food rations, *trans* fatty acids (TFAs) were determined. *Trans* isomers of oleic acid C18:1 *n*-7/*n*-9 were present in the largest quantities, representing from 47% (Day 4) to 73% (Day 2) of all identified TFAs, followed by *trans* isomers of palmitoleic acid C16:1 (8–39%) and *trans* isomers of linoleic acid, C18:2 *tt*, C18:2 *ct* and C18:2 *tc* (6–26%) (Figure 3). The median TFAs content was 0.98% *wt*/*wt* and ranged from 0.50% *wt*/*wt* (Day 10) to 2.11% *wt*/*wt* (Day 9) (Table 2). It should be noted that in over 50% of samples of reconstructed daily food rations analysed (*n* = 6), the TFAs content was below 1% *wt*/*wt*. 

According to experts from the World Health Organization [57], TFAs consumption should not exceed 1% of daily energy intake. The percentage share of TFAs in energy derived from the reconstructed daily food rations ranged from 0.18% (Day 10) to 0.50% (Day 9). Therefore, the TFAs content in the studied daily diets did not exceed the maximum 1% En recommended by the WHO. 

### 3.2. Determination of Nutrients and Fatty Acids Content, Including TFAs, in Breast Milk 

Table 3 presents the results of the nutrient content in grams per 100 mL of breast milk and the energy value in kcal/100 mL of breast milk.

Significant differences were found in individual macronutrient content in breast milk between individual days: 3.1–6.4 g/100 mL for fat; 2.0–5.5 g/100 mL for total protein; 1.6–4.4 g/100 mL for true (nutrition) protein; 1.6–5.6 g/100 mL for carbohydrates (lactose); and 9.4–16.2 g/100 mL for dry matter. The median energy value was 84 kcal (54–95 kcal). Milk from Day 10 (corresponding to Day 9 of maintaining nutrition records) was characterised by the lowest content of nutrients (excluding carbohydrates) and the lowest energy value.

In fat extracted from breast milk samples, 35 fatty acids were identified. The content of selected fatty acids, including TFAs, in breast milk samples, expressed as % *wt*/*wt*, is presented in Table 4. 

Significant differences in the content of both fatty acid groups and individual fatty acids were observed in breast milk between individual days: 40.50–45.24% *wt*/*wt* for SFAs, 36.23–42.75% *wt*/*wt* for MUFAs, 9.74–11.06% *wt*/*wt* for PUFAs, and 0.58–1.13% *wt*/*wt* for TFAs. Among SFAs, myristic (C14:0), palmitic (C18:0) and stearic (C16:0) acids were present in the largest quantities. Oleic acid predominated among MUFAs. Among PUFAs, LA was present in the largest quantities, with the content being over a dozen times higher than that of ALA (6.71–8.30% *wt*/*wt* vs. 0.40–0.68% *wt*/*wt*). The LA-to-ALA ratio was about 13:1. LC-PUFAs of the *n*-3 family and *n*-6 family were present in all analysed breast milk samples. The total *n*-6/*n*-3 fatty acids ratio ranged from about 3:1 to 6:1. In turn, the total SFAs-to-MUFAs-to-PUFAs ratio was 43:40:11.

**Figure 4:**
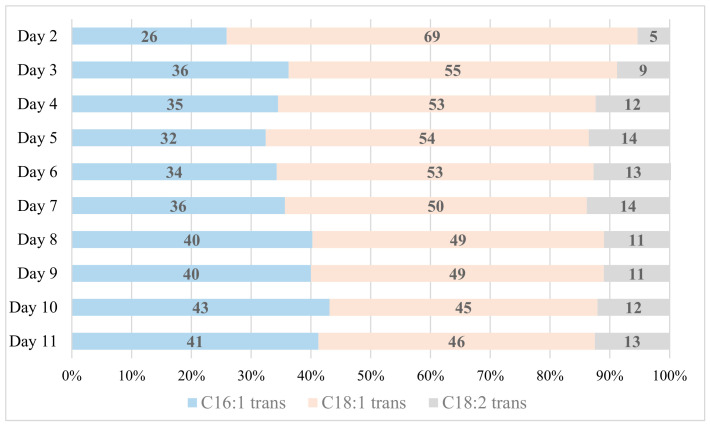
The percentage share of *trans* isomers of individual fatty acids in the pool of all identified *trans* isomers in breast milk samples collected on 10 successive days of lactation.

TFAs were determined in all breast milk samples. *Trans* isomers 18:1 *n*-7/*n*-9 were present in the larger quantity, which represented from 45% (Day 10) to 69% (Day 2) of all identified TFAs (Figure 4), followed by *trans* isomers of palmitoleic acid (C16:1), representing from 26% (Day 2) to 43% (Day 10) of all identified TFAs. *Trans* isomers of linoleic acid, C18:2 *tt*, C18:2 *ct*, and C18:2 *tc,* were also identified. They represented from 5% (Day 2) to 14% (Day 5 and Day 7) of all identified TFAs. The median TFAs content was 0.96% (Table 4). It should be noted that in all tested breast milk samples, the TFAs content was below 2% *wt*/*wt*, ranging from 0.58% *wt*/*wt* (Day 10) to 1.13% *wt*/*wt* (Day 4).

### 3.3. Association Between TFAs Content of Mother’s Diet and TFAs Content in Breast Milk 

**Figure 5:**
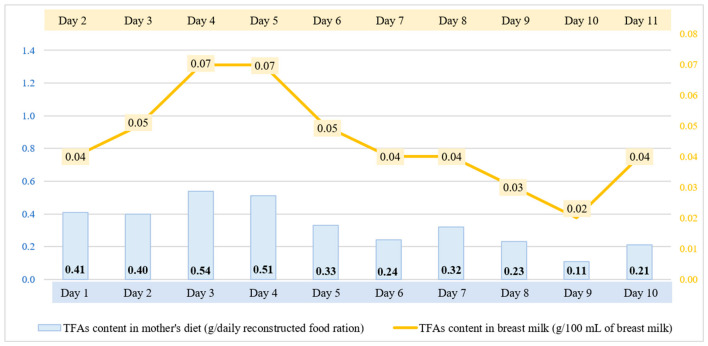
Trajectory of changes in the TFAs content in breast milk of the breastfeeding mother (in g/100 mL of breast milk), in relation to changes in the TFAs content in the daily food ration reconstructed based on the 24 h food consumption survey from the previous day (in g/reconstructed daily food ration).

Figure 5 illustrates the trajectory of changes in TFAs content in the breast milk of the breastfeeding mother (in g/100 mL of milk), in relation to changes in TFAs content in daily food rations reconstructed based on the 24 h food consumption survey from the previous day (in g/reconstructed daily food ration). 

The reconstructed daily food rations were characterised by an average TFAs content of 0.02 g/100 g (range: 0.01–0.03 g/100 g). This corresponded to a value of 0.32 g/reconstructed daily food ration (range: 0.11–0.54 g/reconstructed daily food ration). The median TFAs content in breast milk samples was 0.04 g/100 mL, ranging from 0.02 g/100 mL (Day 10) to 0.07 g/100 mL (Day 4 and Day 5). The increase in TFAs in the diet was associated with an increase in these fatty acids’ content in breast milk, and vice versa. The lowest TFAs intake from the whole-day diet (0.11 g) was reported on Day 9 of the study. The TFAs concentration in breast milk from the next day was 0.02 g/100 mL. On Days 3 and 4 of the study, the TFAs content in the daily food consumed was about 0.5 g, and the TFAs content in samples of breast milk corresponding to those days was 0.07 g/100 mL. With a TFAs content in the diet of 0.2–0.4 g, the TFAs content in breast milk ranged between 0.03 g/100 mL and 0.04 g/100 mL. It should be noted that in all samples of reconstructed daily food rations and of the breast milk analysed, the TFAs content did not exceed 1 g/100 g (for samples of reconstructed daily food rations) and 1 g/100 mL (for breast milk samples).

**Figure 6:**
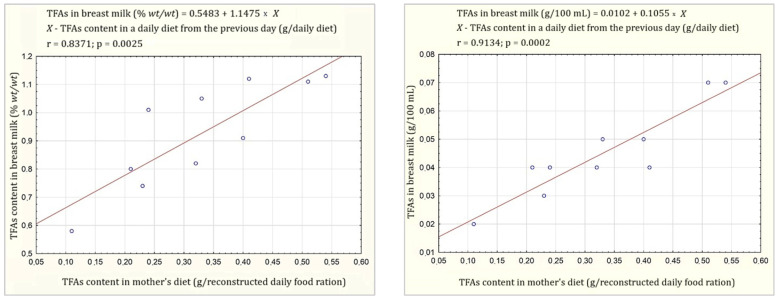
A correlation between the TFAs content in the daily food ration reconstructed based on the 24 h food consumption survey and the TFAs content in breast milk samples collected from a breastfeeding mother from the next day (*n* = 10).

Table 5 and Figure 6 present the results of an analysis of a statistical correlation between the TFAs content in samples of reconstructed daily food rations and the content of those fatty acids in samples of breast milk from the subsequent day. In the conducted statistical analysis, the Pearson linear correlation coefficient (r) and Spearman’s rank correlation coefficient (r_s_) were calculated. 

A positive (r > 0.4, r_s_ > 0.3) correlation was found between the TFAs content in reconstructed daily food rations (g/reconstructed daily food ration) and their levels in breast milk. A statistically strong (*p* < 0.05) correlation was found between the total TFAs content, in g/reconstructed daily food ration and the total TFAs content (in % *wt*/*wt* and in g/100 mL) in breast milk samples (r = 0.84, r_s_ = 0.92 and r = 0.91, r_s_ = 0.86, respectively). A statistically strong (*p* < 0.05) correlation was also found between the total TFAs content (in g/reconstructed daily food ration) and the level of C16:1 *trans* (% *wt*/*wt*) and C18:1 *trans* (% *wt*/*wt*) in breast milk (r = 0.65 and r = 0.78; r_s_ = 0.89, respectively). Similar relationships were obtained after recalculating the content of individual TFAs from % *wt/wt* to g/reconstructed daily food ration. This confirms that the TFAs profile in breast milk depends on the TFAs profile in the diet. In all relations presented above, the Pearson correlation coefficient r and Spearman’s rank correlation coefficient were at least 0.60. 

The equations of linear functions quantitatively describing a functional correlation between the content of total TFAs in the breast milk (% *wt*/*wt*, g/100 mL) and the content of those fatty acids in the breastfeeding mother’s diet (g/daily diet) from the previous day are as follows:

$$\begin{array}{c} \mathrm{TFAs}\;\mathrm{in}\;\mathrm{breast}\;\mathrm{milk}\;(\%\;wt/wt)\;=\;0.5483\;+\;1.1475\times X\\ \mathrm{or}\\ \mathrm{TFAs}\;\mathrm{in}\;\mathrm{breast}\;\mathrm{milk}\;(g/100\;\mathrm{mL})\;=\;0.0102\;+\;0.1055\times X \end{array}$$

where *X* is the TFAs content in the daily diet from the previous day (g/daily diet).

The results of the present analysis also showed that the content of *trans* isomers of oleic acid in the diet (% *wt*/*wt*) does not have a statistically significant effect on the TFAs content in breast milk, even though they are the main *trans* isomers in breast milk. However, after recalculating the dietary C18:1 *trans* content from % *wt*/*wt* to g/reconstructed daily food ration, a statistically significant positive correlation was observed with the total TFAs content in breast milk (in % *wt*/*wt*, g/100 mL). The Pearson correlation coefficient r and Spearman’s rank correlation coefficient were above 0.60. In contrast, *trans* isomers C16:1 (in % *wt*/*wt* and in g/reconstructed daily food ration) and *trans* isomers C18:2 (in % *wt*/*wt* and in g/reconstructed daily food ration) from the diet are statistically significantly correlated with the total TFAs content in breast milk. 

Due to the small number of samples, the data described above should be interpreted with great caution.

## 4. Discussion

According to our best knowledge, this is the first study of this type—in which the analytically determined TFAs content in breast milk was compared to the analytically determined content of those fatty acids in samples of daily food rations reconstructed based on questionnaires of the 24 h food consumption survey from the previous day. For this purpose, 10 reconstructed daily food rations from one breastfeeding mother and samples of her breast milk were used.

The diet of the studied breastfeeding woman was varied. It contained products from many groups, including milk and dairy products, meat and meat products, cereal products, eggs, vegetables and fruit, as well as confectionery and bakery products and fast food products. On the other hand, despite the recommendations, the study participant’s diet did not include fish and fish products, while beverages containing caffeine (coffee) were consumed every day. Furthermore, the weight of the daily food consumed by the study participant was alarmingly low (Me = 1664.4 g; range: 638.0–2355.0 g). We found that the subject’s diet significantly differed from the recommendations specified for this group. In this analysis, norms from 2020 developed by NIPH NIH-NRI were used as a standard [58]. The energy value of all 10 daily diets analysed was also alarmingly lower than recommended (Me = 972 kcal/day; range: 198–1944 kcal/day). The percentage share of the daily energy intake from fat ranged from 23% (Day 9) to 41% (Day 7) and exceeded the national recommendations for breastfeeding women (norm: 20–35% of energy) in 50% of analysed daily diets (Days 1, 4, 5, 7 and 10). The percentage share of the daily energy intake from other nutrients was, on average, 17%En (12–25%En) for protein, 46%En (41–53%En) for carbohydrates, and 2%En (1–4%En) for dietary fibre. Considering the above, it should be emphasised that regardless of whether nutritional habits during lactation significantly influence the content of individual nutrients in breast milk, the nutritional status of a breastfeeding woman is of importance for her health. The diet deviating from the recommended norms, either a deficiency or a poorly balanced diet, may lead, e.g., to decreased metabolism, weakening and poorer concentration. 

The fatty acids content in reconstructed daily food rations varied widely between individual days: 42.67–65.47% *wt*/*wt* for SFAs, 29.15–43.89% *wt*/*wt* for MUFAs, 3.25–14.65% *wt*/*wt* for PUFAs, and 0.50–2.11% *wt*/*wt* for TFAs. The energy percentage from SFAs was 18% on average and ranged from nearly 15% (Days 4, 6, and 9) to 22% (Day 7). In all daily diets, the SFAs consumption exceeded the WHO recommended daily intake of no more than 10% of the energy [57]. The consumption of EPA + DHA (Me = 4 mg, range: 2–44 mg) and DHA (Me = 2 mg, range: 1–40 mg) was below recommended levels. The EPA + DHA and DHA intake in the diet of the studied woman covered the recommended daily intake of those fatty acids in 1–17% (EPA + DHA) and 1–20% (DHA). The percentage share of energy from LA and ALA ranged between 0.6 and 4.9%En, and 0.1 and 0.7%En, respectively, and in the majority of evaluated daily rations it was below the norm (minimum of 4%En for LA and of 0.5%En for ALA). The total *n*-6/*n*-3 fatty acids ratio also differed from the beneficial ratio (about 2:1), ranging between about 6:1 and 25:1. For healthy infant development during the breastfeeding period, SFAs consumption should be reduced, while LC-PUFAs intake should be increased. Comparing the obtained data to the results of our previous studies on daily hospital diets for pregnant and breastfeeding women [55], we found lower average values for weights of daily food rations (1664.4 g vs. 2866.7 g), energy (972 kcal vs. 2176 kcal), and fat content (35.8 g vs. 67.9 g). Some studies suggest that eating disorders intensifying in the postpartum period result from the aesthetic pressure and the wish to lose weight quickly by eating less calories than required [59]. In our study, the participant’s insufficient dietary intake of the recommended amounts of energy and nutrients could result, on the one side, from the lack of time to prepare and eat a meal (the study participant was a mother of three children: twins who were breastfed and an older child aged six). On the other side, this could result from the daily consumption of white coffee (at least 300 mL/day). The results of our findings suggest a need for rapid intensification of educational activities addressed to breastfeeding women. Also, Carretero-Krug et al. [60] in their work reviewing reports on the nutritional status of breastfeeding mothers and the influence of diet on breast milk composition indicate that in the breastfeeding period attention is mainly focused on the newborn baby, while surprisingly little attention is dedicated to the diet of the breastfeeding mother. Considering the above, providing support from qualified dietary personnel during the first 6 months of the infant’s life appears to be of particular significance, as during that time the mother’s milk should be the exclusive and basic food of the child.

*Trans* fatty acids were identified in all samples of reconstructed daily food rations. *Trans* isomers of oleic acid were the most abundant (47–73%). *Trans* isomers C16:1 (8–39%) and *trans* isomers C18:2 *n*-6 (6–26%) were present in lower quantities. The data obtained in this study are similar to the results of our studies from 2022 [55], in which the highest share (62% to 68%) of *trans* isomers C18:1 *n*-7/*n*-9 were found in the pool of all TFAs identified in daily hospital diets for pregnant and breastfeeding women. *Trans* isomers C18:2 and C16:1 represented from 20% to 24% and from 9% to 18%, respectively, of all identified TFAs. In the work from 2022 cited above, we found that the greatest quantities of TFAs were introduced into the hospital diet by dairy products containing r-TFAs. Having data on the weight of ingredients in every meal, we attempted to estimate the share of products being a source of r-TFAs (group 1: milk and dairy products and meat and meat products) and of i-TFAs (group 2: bakery products, confectionery products and biscuits, salad sauce concentrates, and fast food products) in the total weight of the currently evaluated daily food rations. The share of products from the first group in the study participant’s daily diet ranged from 21% (Day 4) to 51% (Day 7). On the other hand, the share of products from the second group was several to several dozen times lower and ranged from 0% (Days 5 and 9) to 20% (Day 6). It appears that the TFAs identified in the currently analysed diet of the breastfeeding mother derived mainly from milk and dairy products and from meat and meat products. We noted that the TFAs intake in the diet of the breastfeeding woman covered by this study ranged from 0.50% *wt*/*wt* to 2.11% *wt*/*wt* (0.11–0.54 g/reconstructed daily food ration) and was lower than that estimated previously: 10–14 g/person/day in 1995 [61], 3.3 g/person/day in 1998 [62], 2.8 g/person/day in 1999 [63], or 0.72 g/daily hospital diet in 2022 [55]. The percentage share of energy from TFAs in the currently evaluated daily food rations ranged from 0.18 to 0.50%, so it did not exceed 1% of energy from the diet and corresponded to the WHO recommendations [57]. 

Despite the deficient diet of the breastfeeding mother analysed in this study, the nutrient content in her milk was comparable on individual days. At the same time, we found that the average fat content (4.9 g/100 mL) was higher than noted in earlier studies for human breast milk (from Day 10 to Day 24 of lactation) from Polish women (2.9–4.4 g/100 mL) [64,65,66,67,68,69]. The average content of total protein (4.8 g/100 mL), dry matter (13.7 g/100 mL) and energy (84 kcal/100 mL) of the currently studied breast milk samples was also higher than that found in the studies cited above (total protein: 1.0–1.7 g/100 mL; dry matter: 12.5–13.45 g/100 mL; energy: 60.0–77 kcal/100 mL). In turn, the average carbohydrate content in the milk of breastfeeding women from Poland found in previous studies ranged from 7.1 to 8.10 g/100 mL and was therefore higher than in the milk of the breastfeeding mother from this study (3.8 g/100 mL). It should be noted that breast milk composition may change depending, e.g., on changes in the diet [65,70] and nutrition status of the breastfeeding mother [71]. It appears that the mother’s mental state may also influence the composition of breast milk [72]. Additionally, some studies suggest that macronutrient content in breast milk may be adjusted to the child’s sex; however, the extent of this effect is not fully known. In recently published works, it was demonstrated that milk produced for boys is richer in proteins, fats, and carbohydrates, so it is characterised by higher energy compared to milk produced for girls [73,74]. Fumeaux et al. [75] and Thakkar et al. [76] in their earlier studies also demonstrated the effect of male sex of the child on the fat content and energy in breast milk. On the other hand, Bzikowska-Jura et al. [77] noted that human milk collected from mothers of male infants was characterised by a statistically significantly higher content only of carbohydrates compared to the milk of mothers of female infants (7.13 g/100 mL vs. 6.95 g/100 mL). In contrast to the studies cited above, Fujita et al. [78] observed a relationship between female sex of the child and higher fat content in breast milk. With regard to protein and carbohydrates, the literature indicates the influence of various factors on their content in breast milk, including the mother’s BMI before pregnancy and during lactation, anthropometric parameters of the child [79,80,81] and child’s sex, number of feedings a day, and child’s birth weight [77], and the volume of produced milk [82,83]. Additionally, according to Hahn et al. [84], lower levels of carbohydrates in breast milk were associated with a caesarean section. It should be noted that in the studies cited above on the influence of selected factors on the macronutrient content in breast milk, the research included mothers that bore one child. We still do not know how multiple pregnancy influences the macronutrient content in breast milk. On the other hand, in several studies, the macronutrient content was determined in the milk of mothers using tandem breastfeeding, i.e., feeding two children of different ages during the same period [85,86,87,88]. However, the data obtained in these studies, as in the case of the effect of the child’s sex on milk composition, did not yield clear-cut results. For example, in the study by Cinar et al. [85], no effect of tandem breastfeeding on macronutrient content in breast milk was found. On the other hand, Sinkiewicz-Darol et al. [87] noted a higher average concentration of fat (4.2 g/100 mL), total protein (1.1 g/100 mL), true protein (0.9 g/100 mL), dry matter (12.6 g/100 mL), and energy (72.4 kcal/100 mL) in breast milk samples from tandem breastfeeding compared to samples collected after weaning the older child (3.4 g, 0.9 g, 0.8 g, 11.6 g, 63.4 kcal, respectively). The authors of the cited study pointed out that younger children have increased nutritional needs. Therefore, it can be suspected that the fat, protein, and dry matter content and, in consequence, higher energy of the studied woman’s milk noted in this study, compared to the previously cited literature data, are the result of her milk’s composition adapting to the nutritional needs of twins of different sexes. On the other hand, the carbohydrate content is lower than that found in earlier studies and may be associated with the birth type (caesarean section), and this was confirmed by Hahn et al. [84]. Because the composition of human milk is highly dynamic and sensitive to external factors, undoubtedly these data should be validated in a larger group of study participants.

In fat extracted from breast milk samples, we found over 30 different fatty acids, the content of which varied significantly: 40.50–45.24% *wt*/*wt* for SFAs, 36.23–42.75% *wt*/*wt* for MUFAs, and 9.74–11.06% *wt*/*wt* for PUFAs. *Trans* fatty acids were identified in each breast milk sample. Their level was lower than in earlier studies. In this study, the value was 0.96% *wt*/*wt* (range: 0.58–1.13% *wt*/*wt*; 0.02– 0.07 g/100 mL), versus 2.59% *wt*/*wt* (1.49–3.34% *wt*/*wt*) for milk in lactation week 5–6 and 2.36% *wt*/*wt* (1.55–3.92% *wt*/*wt*) in lactation week 9–10 in spring, and 2.41% *wt*/*wt* (1.79–4.31% *wt*/*wt*) in lactation week 5–6 and 2.77% *wt*/*wt* (1.53–4.18% *wt*/*wt*) in lactation week 9–10 in autumn, in 2003 [29]; 2.49–1.01% *wt*/*wt* on lactation Day 90 [28] in 2011; or 2.42% *wt*/*wt* for transitional milk and 2.28% *wt*/*wt* for mature milk in 2024 [30]. Among the TFAs, *trans* isomers C18:1 (Me = 0.51% *wt*/*wt*) were present in the greatest quantity in the current study. *Trans* isomers C16:1 (Me = 0.33% *wt*/*wt*) and C18:2 (Me = 0.10% *wt*/*wt*) were also identified. Additionally, the wide ranges of min–max values for the content of C18:1 *trans* isomers (0.26–0.77% *wt*/*wt*) found in this study suggest a significant variation in the intake of these fatty acids in the diet. On the other hand, narrow min–max ranges for *trans* isomers C16:1 (0.25–0.39% *wt*/*wt*) and C18:2 (0.06–0.15% *wt*/*wt*) acids indicate their constant and similar intake in the diet. The results of our study are comparable with the results of earlier studies, in which *trans* isomers C18:1, C18:2 and C16:1 fatty acids were also indicated as the most abundant group of TFAs in the milk of women from Poland. However, Mojska et al. [29]’s findings in the mature milk (lactation week 9–10) of women from Mazowieckie Voivodeship indicate higher average values for *trans* isomers C18:1 (1.98% *wt*/*wt* in spring, 2.43% *wt*/*wt* in autumn), similar values for *trans* isomers C16:1 (0.31% *wt*/*wt* in spring, 0.37% *wt*/*wt* in autumn) and lower values of *trans* isomers C18:2 (0.00% *wt*/*wt* in spring and autumn). Also, Martysiak-Żurowska et al. [28] in milk samples collected at 90 day of lactation from women from Gdańsk and its region reported higher average values for *trans* isomers C18:1 (1.22% *wt*/*wt*). For *trans* isomers C16:1 and C18:2, the cited authors noted values that were higher (0.50% *wt*/*wt*) and similar (0.08% *wt*/*wt*) to those presented in this study, respectively. On the other hand, Purkiewicz and Pietrzak-Fiećko [30] indicate lower average values of *trans* isomers C16:1 in the mature milk (0.09 ± 0.07% *wt*/*wt*) of Polish women living in Warmia and Mazury provinces. For *trans* isomers C18:1 and C18:2 in mature milk samples, the cited authors identified vaccenic acid (C18:1 *trans*-11)—1.52 ± 1.26% *wt*/*wt*; petroselaidic acid and elaidic acid (C18:1 *n*-6 + *n*-9 *trans*)—0.49 ± 0.25% *wt*/*wt*; and rumenic acid (C18:2 *cis*-9 *trans*-11)—0.18 ± 0.29% *wt*/*wt*. At the same time, the authors of the study cited above noted that the TFA predominating in colostrum, transitional milk, and mature milk was vaccenic acid, which is characteristic for animal fats. 

Low total food intake was associated with low dietary intake of TFAs and, as a result, affected the concentration of TFAs in milk. However, TFAs content above the limit of quantification was identified in every milk sample (LOQ = 0.01% *wt*/*wt*). We found a statistically significant linear correlation between the TFAs content in samples of daily food rations (g/daily food ration) and TFAs levels in the breast milk samples (% *wt*/*wt* and g/100 mL) from the next day (r = 0.84, r_s_ = 0.92 and r = 0.91, r_s_ = 0.86, respectively). These correlations are described by function formulas that appear to be usable for calculating the TFAs content in breast milk based on the TFAs content in the whole-day diet from the previous day: ƩTFAs in breast milk (% *wt*/*wt*) = 0.5483 + 1.1475 × ƩTFAs in the daily diet from the previous day (g/daily diet) or ƩTFAs in breast milk (g/100 mL) = 0.0102 + 0.1055 × ƩTFAs in the daily diet from the previous day (g/daily diet). The proposed equations to preliminarily explore the linear relationship between these parameters should be considered with caution and with necessary interpretational reserve. Undoubtedly, they should be validated in a larger group of study participants. It should be noted that the available literature provides several other formulas, which may be used to estimate dietary TFAs intake based on the analytically determined TFAs content in breast milk. However, these methods use the content of *trans* isomers C18:1 in breast milk as a “reflection” of their content in a breastfeeding mother’s diet from the previous day [48] or the TFAs content in the adipose tissue, which reflect long-term TFAs intake [89,90,91]. Moreover, Craig-Schmidt’s formula [89] can be used to estimate short-term TFAs intake in breastfeeding women’s diets only when *trans* C18:1 content in breast milk exceeds 1.49% *wt*/*wt*. Taking into account the trend of TFAs (mainly *trans* C18:1) content decreasing in food and breast milk, the use of the above formula poses a potential risk of under- or overestimating the TFAs content in milk. Bousset-Alféres et al. [25] noted that the elaidic acid content in transitional and mature milk, estimated with Craig-Schmidt’s formula, was significantly higher (transitional milk: 1.76%; mature milk: 1.78%) compared to their own results (transitional milk: 0.50%; mature milk: 0.58%). Our study was conducted after legislation limiting the i-TFAs content in food came into force in Poland (below 2 g/100 g of fat) [92]. Additionally, no commercial products containing partly hydrogenated oils/fats were found in the diet of the studied person. Therefore, it seems that the formula proposed by us, quantitatively determining the relationship between the TFAs content in their milk and in the diet, can be used in currently conducted studies in this area.

The results of this study also showed that the content of *trans* isomers of oleic acid in the diet (in % *wt*/*wt*) does not influence the TFAs content in breast milk in a statistically significant manner, although they are the main *trans* isomers in breast milk. But after recalculating the content from % *wt*/*wt* to g/reconstructed daily food ration, a statistically significant positive correlation was observed with the total TFAs content in breast milk (in % *wt*/*wt*, g/100 mL). In contrast, *trans* isomers C16:1 and C18:2 (in % *wt*/*wt* and in g/reconstructed daily food ration) are statistically significantly correlated with the total TFAs content in breast milk. This is probably associated with the fact that the human body can differentiate between individual TFAs by incorporating these compounds into human cells via a specific mechanism [93]. According to Moore and Dhopeshwarfar [94], elaidic acid (*trans* isomer of oleic acid, C18:1) is preferentially incorporated into human cells. Our study takes into account the total TFAs content in the mother’s diet and breast milk and, at the same time, confirms the influence of the short-term dietary intake of total TFAs on the content of these fatty acids in breast milk. Similar relationships have been reported in several other studies [25,29,33,34,35,36,37,38,39]. According to Risé et al. [95], short-term dietary intake of fatty acids, including TFAs, is best reflected by measuring their levels in body fluids (breast milk and serum), because the composition of this medium is considered representative of the recent fat intake. Furthermore, studies conducted by Aitchison et al. [32] and Craig-Shmidt et al. [48] show that fatty acids from the mother’s diet may be present in her breast milk within 12–36 h after a meal, although changes in the fatty acids composition often reflect the diet from the last 1–3 days. In contrast to the studies cited above, Zielińska-Pukos et al. [39], Bousset-Alféres et al. [25], Samur et al. [33], Aumeistere et al. [37], and Daud et al. [34] did not find a statistically significant correlation between the TFAs content in the diets of studied women—as estimated based on dietary interviews—and the concentration of these fatty acids in breast milk. According to Butts et al. [36], the lack of consistency in assessing the correlation between the aforementioned parameters stems, among others, from complex metabolic co-dependencies between fatty acids in the diet and milk. It should also be emphasised that in many studies, single samples of breast milk were collected on the same day, based on which nutritional information was recorded. According to Yang et al. [96], using such a milk sample instead of a 24 h composite sample may affect the assessment of the relationships between maternal factors and breast milk composition and, therefore, negatively affect the representative character of a study. At the same time, it should be noted that the TFAs profile in food is varied, and the content of individual TFAs and their total amount can be influenced by many factors [97,98]. With no comprehensive, and especially current, data about the TFAs content in food products available on the market in a given country, incomplete data or databases from other countries are used to estimate the TFAs intake in a diet. The use of this approach may result in underestimation of the total TFAs content in a diet, at the same time affecting the interpretation of the study results. For example, Aumeistere et al. [37] used the Finnish database Fineli to estimate the TFAs intake in a breastfeeding Latvian women’s diet. The authors of the study did not find a statistically significant correlation between the TFAs content in the diet and the TFAs level in breast milk, stating as a reason the lack of national data on the TFAs content in food products. Thus, a determination of a formula quantitatively determining a relationship between TFAs intake in a diet and the level of these fatty acids in milk seems to be even more important in view of current changes in the TFAs content in food.

The results of our study confirm a positive change in the TFAs content over the last more than 20 years, showing a decrease in the i-TFAs content in food and diets worldwide [99]. The effective reduction in i-TFAs in food was achieved by a combination of voluntary (e.g., manufacturers’ actions to reform composition of their products) and regulatory (e.g., i-TFAs limits in food and bans on the use of PHOs) strategies [49,50,100]. Interesting results were provided by a study conducted by Ratnayake et al. [46] which aimed to analyse the TFAs content in the milk of Canadian women before and after introduction of legislative regulations. The authors of that work noted that high TFAs levels determined in breast milk before legislative regulations coming into force (7.2 ± 0.3% *wt*/*wt* in 1992) were significantly decreased following their coming into force (1.9 ± 0.3% *wt*/*wt* in 2011). Also, Friesen and Innis [54], comparing the TFAs level in milk collected from women in 2004–2005 to the level of these fatty acids in milk collected from women in 1998, noted a decrease in the TFAs content from 7.1 ± 0.32% *wt*/*wt* in 1998 (*n* = 103), through 6.2 ± 0.48% *wt*/*wt* and 5.3 ± 0.49% *wt*/*wt* in 2004–2005 (*n* = 48), to 4.6 ± 0.32% *wt*/*wt* in 2006 (*n* = 39). According to the authors of the studies cited above, a decrease in the TFAs content in breast milk reflects a change in the intake of these fatty acids in the diet of Canadian breastfeeding mothers, resulting from introduction of national regulations regarding i-TFAs. Data presented by WHO [101] show that by the end of 2023, in 53 countries (including Poland) policies based on the best practices of eliminating harmful *trans* fats were in force, covering 3.7 billion people, i.e., 46% of the global population, versus 6% 5 years earlier. 

Based on the data from the studies cited above and the results of this study, it should be noted that in recent years, the profile of fatty acids intake has also changed. Until recently, elaidic acid (C18:1 delta-9 *trans*) was the most abundant TFA in the diet, and therefore in breast milk, which is also considered as a main *trans* isomer of fatty acids of industrial origin. Therefore, in earlier studies, products containing partially hydrogenated plant oils (source of i-TFAs), for example, margarine, bakery products, snacks, confectionery products and biscuits, and fried food, were indicated as the main source of TFAs in mothers’ diets [28,29,33,34,42,47,102,103]. Thus, in earlier studies in this area, the use of Craig-Schmidt’s formula [48] appears to have been appropriate to estimate the TFAs intake within breastfeeding women’s diets based on *trans* C18:1 levels in breast milk. As the i-TFAs content in food was significantly reduced, and r-TFAs levels in milk, meat, and their products has been relatively stable for many years [104], currently the main source of *trans* isomers C18:1 in breastfeeding women’s diets, and in consequence in breast milk, appears to be products that are a natural source of TFAs. Especially, national recommendations for lactating women recommend daily consumption of 2–3 servings (glasses) of natural milk products, preferably fermented ones [105,106]. Also, Aumeistere et al. [38] observed that the TFAs level in the milk of Latvian women was associated with customary consumption of meat and meat products (ρ = 0.296, *p* = 0.021) and of milk and milk products (ρ = 0.566, *p* < 0.0005). Similar relationships were also noted in Croatian [103], Brazilian [107], and Slovenian [108] breastfeeding mothers. According to Zupanič et al. [108], due to the reduction in i-TFAs in Slovenian food, r-TFAs derived from milk, meat, and their products currently represent the largest percentage of TFAs intake in this country. The influence of the level of milk and dairy product consumption on TFAs levels in breast milk was also confirmed in a study by Martysiak-Żurowska et al. [109]. The aim of that study was to compare the composition of milk from 30 women on a diet with unlimited consumption of milk and its products (>12.7 ± 0.7 g of milk fat/day) to the milk of 20 women on a diet poor in milk and dairy products (<4.2 ± 0.5 g of milk fat/day). The authors of the cited study found a statistically significantly higher content of vaccenic acid in the milk of women from the first group (0.69% *wt*/*wt*) compared to the milk of women included in the group with low milk and dairy product consumption (0.36% *wt*/*wt*). Therefore, it seems that a reduction in consumption of products containing i-TFAs during lactation does not guarantee low TFAs levels in milk. 

In conclusion, it should be noted that this study provides a comprehensive evaluation of energy and macronutrient content, with a particular emphasis on fatty acids, including TFAs, in the milk of a mother of twins of different sexes, in comparison to the composition of her diet, assessed based on analytical testing of reconstructed daily food rations. At the same time, it should be noted that the samples of daily food rations and breast milk came from only one breastfeeding mother. Therefore, the presented results should be treated as pilot ones, and the research should be continued.

## 5. Limitations

The only limitation of our study is that it was conducted with one participant. The statistical analysis was based on a sample consisting of 10 observations. Therefore, the statistical strength of the tests is limited—with such a small sample the risk of type 2 error (failure to reject a null hypothesis) is higher. Furthermore, correlation coefficients may be unstable—a single observation may significantly influence the coefficient value. Thus, the results obtained in this study should be interpreted with caution.

However, our study included whole-day breast milk samples collected after completing the 24 h food consumption survey. The methods used to collect milk samples and the storage conditions were appropriate. The literature most often describes single-day human milk samples, which are collected on the same day as the dietary record. According to Yang et al. [95], the use of single breast milk samples collected on the same day that dietary habits were noted, instead of a composite 24 h milk sample, may affect the assessment of the relationships between maternal factors and breast milk composition. This may therefore negatively impact the representative character of a study. 

Secondly, milk samples were collected from a mother breastfeeding twins of different sexes over 10 consecutive days. To our best knowledge, this is the first study in which the energy value, macronutrients and fatty acids content, including TFAs, were determined over 10 consecutive days in the human milk of a mother breastfeeding twins of different sexes. The observed surprising variations in the macronutrient content in the breast milk of the study participant need to be confirmed in further studies with a larger number of participants, especially that the composition of the human milk is variable within a feeding, diurnally, over lactation, and between mothers. However, our study may provide a basis for further research in this field and for increasing knowledge on this subject.

Thirdly, the obtained results for the TFAs content in the breast milk samples were presented as g/100 mL of milk, and this allows for an assessment of the intake of these compounds by infants. In the majority of studies in this area, a result of the TFAs content in the tested samples was provided as % *wt*/*wt*. When no results for fat content were provided, it was not possible to evaluate the intake of these fatty acids by breastfed infants. 

Fourthly, our study covers the 10-day diet of a mother breastfeeding twins of different sexes. To determine the nutritional value of the diet, ten reconstructed daily food rations were used. It should be noted that the 3-day food intake record is the most frequently described method in the literature. Therefore, our study is the first that provides a comprehensive 10-day assessment of energy value, macronutrients, and fatty acids content, including TFAs, in the diet of a mother breastfeeding twins of different sexes. The strength of our study is that the study participant used a digital kitchen scale to record the weight of servings of dishes and products consumed during completion of the survey questionnaire. Additionally, the name and brand of each commercial product was noted. Furthermore, the study participant described in detail the cooking techniques used to prepare her meals, like frying and cooking times and the quantity of oil used to prepare her meals. The weight of stone fruit included only the eatable parts (with the stone removed). Thus, reconstruction of daily food rations in laboratory conditions was unaffected by errors related to weighing incorrect quantities of products or dishes. The wide ranges in energy value and macronutrient content of the study participant’s diet are most likely due to the fact that, after childbirth, women seem to focus primarily on the health of their newborns rather than their own and have little time to prepare meals for themselves. Insufficient intake of the recommended amounts of energy and nutrients is quite common. The participant of our study was not only the mother of twins whom she was breastfeeding, but she also had an older child aged six. It is therefore no surprise that during those 10 days, there were days when she consumed very small amounts of food. Such situations may be missed in a 24 h dietary recall—even a repeated one—or a food frequency questionnaire. Recording the mother’s diet for 10 consecutive days makes it possible to identify these situations. Undoubtedly, these data should be validated in a larger group of participants. However, our study may be a starting point for further research in this field.

Fifth, this is the first study in which we have developed equations to preliminarily explore the linear relationship between the TFAs content in breast milk and the TFAs content in the whole-day diet from the previous day. It should be noted that the available literature provides inconsistent data on the effect of dietary TFAs intake on their content in the breast milk. Most often, this is an effect of under- or overestimated dietary TFAs intake. In addition, the decrease in the TFAs content in food observed recently seems to make it impossible to use the existing Craig-Schmidt’s formula to estimate the TFAs content in the diet or in breast milk. Our study is the first to propose new formulas for the preliminary calculation of TFAs content in the diet and in breast milk.

Sixth, the advantages of this study include the use of modern analytical techniques. TFAs content results in the daily diet and breast milk were obtained by gas chromatography–mass spectrometry in a laboratory that uses internal and external methods of quality evaluation. This work is also remarkable for the use of two statistical tests that allowed determination of which parameter contributed to the change in the fatty acids profile in breast milk to the greatest extent. In the statistical analysis, Pearson’s and Spearman’s correlation coefficients were calculated. The simultaneous use of both coefficients provides a more complete understanding of the relationship between variables, especially when there are doubts about whether the assumptions of parametric analysis are met or when the sample size is limited.

In general, to our best knowledge, this is the first study that provides information on TFAs intake with the 10-day diet of a breastfeeding mother of twins, based on laboratory-measured content of these compounds in breast milk samples, and in daily dietary rations reconstructed using data from 24 h food consumption surveys. The data on the mother’s food intake provided detailed, quantitative and descriptive perspectives about the nutritional practices of the mother of twins after their birth. The study should be repeated with a larger number of participants. 

## 6. Conclusions

In our study, we evaluated the energy value, macronutrient and fatty acids, including TFAs, content in the diet and breast milk of a mother breastfeeding twins of different sexes. For this purpose, 10 daily food rations from the breastfeeding mother and samples of her breast milk were used. 

According to our knowledge, this is the first study in which a 10-day diet of a breastfeeding mother of twins—reconstructed based on a 24 h food consumption survey—was correlated with *trans* fatty acids (TFAs) concentrations in breast milk. We observed dietary-dependent fluctuations in the TFAs content in the breast milk. This single-participant pilot study suggests that the TFAs content in breast milk may be a marker of the dietary intake of these compounds from the previous day. Based on the correlation between the TFAs content in the breastfeeding mother’s diet and the concentration of these fatty acids in the breast milk, we have developed an equation to preliminarily explore the linear relationship between these parameters. These hypothesis-generating equations could be used to calculate the TFAs content in the breast milk based on the TFAs content in the whole-day diet from previous day. This is important because it appears that the formula proposed by Craig-Schmidt et al. in the mid-1990s can no longer be used in such studies due to the decreasing trend in TFAs content in food products and breast milk observed in recent years. Currently, it seems the level of TFAs in breast milk depends on the consumption of milk, meat, and their products. Due to the small number of samples, data from this study should be interpreted very cautiously and validated in a larger cohort.

It seems that our single-participant pilot study highlighted differences in the dietary practices of a mother of twins. It provides a basis for further research in this area. The analysis of selected parameters of the nutritional value of the study participant’s diet provided alarming results. The evaluated food rations provided too many SFAs and too few PUFAs compared to the recommended norms. The energy of daily food rations and their total weight were also lower than recommended. The wide ranges in energy value and macronutrient content in the study participants’ diets are most likely due to the fact that, after childbirth, women seem to focus primarily on the health of their newborns rather than their own. The surprising changes in the macronutrient content in milk over the 10 consecutive days observed in this study also need to be confirmed in further research.

Our study emphasises the importance of an analysis of individual food consumption by a breastfeeding mother of twins on her milk composition, including TFAs content. This is a useful source of information about implementing dietary recommendations, as it provides new information about dietary patterns during the lactation period and, consequently, the impact of the mother’s diet on milk composition. Therefore, it can be suspected that the development of personalised nutritional strategies will significantly influence infants’ health.

## Figures and Tables

**Table 1:** Comparison of the weight (g), nutrient content (g), and energy (kcal) of reconstructed daily food rations.

Parameter	Day 1	Day 2	Day 3	Day 4	Day 5	Day 6	Day 7	Day 8	Day 9	Day 10	Days 1–10 Median (Min–Max)
**Reconstructed daily food ration** **weight** (g)	2262.0	2355.0	1834.2	1734.0	1682.2	800.0	922.0	1098.0	638.0	1646.5	1664.4 (638.0–2355.0)
**Fat content in the reconstructed** **daily food ration** (g/reconstructed daily food ration)	79.2	30.6	34.8	52.0	28.6	36.8	47.9	24.2	5.1	42.8	35.8 (5.1–79.2)
**Protein content in the reconstructed** **daily food ration** (g/reconstructed daily food ration)	73.7	43.1	40.0	40.7	28.4	46.4	31.7	23.2	12.2	45.8	40.4 (12.2–73.7)
**Carbohydrates content in the reconstructed daily food ration** (g/reconstructed daily food ration)	244.3	103.4	133.5	178.9	68.8	115.3	129.4	92.0	26.2	123.3	119.3 (26.2–244.3)
**Fibre content in the reconstructed** **daily food ration** (g/reconstructed daily food ration)	20.4	9.4	11.0	24.3	2.5	9.6	11.1	4.4	1.0	8.2	9.5 (1.0–24.3)
**Energy of reconstructed daily** **food ration** (kcal/reconstructed daily food ration)	1944	843	986	1298	641	959	1054	669	198	1045	972 (198–1944)

**Table 2:** Comparison of selected fatty acids content, including TFAs (% *wt*/*wt*) * in the reconstructed daily food rations.

Fatty Acids	Day 1	Day 2	Day 3	Day 4	Day 5	Day 6	Day 7	Day 8	Day 9	Day 10	Days 1–10 Median (Min–Max)
**Total Saturated Fatty Acids (SFAs)** [% *wt*/*wt*]											
C14:0 *(Myristic Acid)*	3.02	8.36	10.01	4.38	7.53	3.87	5.19	9.06	10.60	5.89	6.71 (3.02–10.60)
C16:0 *(Palmitic Acid)*	23.54	29.77	31.93	25.81	27.20	23.42	29.93	24.72	32.36	23.91	26.51 (23.42–32.36)
C18:0 *(Stearic Acid)*	13.63	11.34	10.25	8.82	9.04	12.37	9.88	11.31	13.03	10.61	10.96 (8.82–13.63)
Total SFAs	42.98	56.25	62.16	42.67	50.76	42.98	53.04	60.45	65.47	50.31	51.90 (42.67–65.47)
***cis*-Monounsaturated Fatty Acids (MUFAs)** [% *wt*/*wt*]											
C16:1 *cis (cis-Palmitoleic Acid)*	1.49	1.79	1.40	1.41	3.58	1.69	0.93	0.95	1.90	1.06	1.45 (0.93–3.58)
C18:1 *cis (cis-Oleic Acid)*	41.80	31.64	27.88	39.60	30.25	39.18	35.03	28.32	25.70	36.33	33.34 (25.70–41.80)
Total MUFAs	43.89	34.58	30.32	41.65	34.98	41.96	36.59	30.14	29.15	38.14	35.79 (29.15–43.89)
***cis*-Polyunsaturated Fatty Acids (PUFAs)** [% *wt*/*wt*]											
C18:3 *n*-3 (*α-Linolenic Acid*, ALA)	1.15	0.82	0.51	0.88	0.49	1.92	0.71	1.01	0.24	1.58	0.85 (0.24–1.92)
C20:5 *n*-3 (*Eicosapentaenoic Acid*, EPA)	<LOQ **	<LOQ **	<LOQ **	<LOQ **	<LOQ **	<LOQ **	<LOQ **	<LOQ **	<LOQ **	<LOQ **	0.01 (<LOQ **–0.01)
C22:6 *n*-3 (*Docosahexaenoic Acid*, DHA)	0.05	<LOQ **	<LOQ **	0.06	<LOQ **	<LOQ **	<LOQ **	0.01	0.03	0.01	0.01 (<LOQ **–0.06)
Total LC-PUFAs *n*-3	1.22	0.83	0.51	1.01	0.50	1.99	0.87	1.03	0.29	1.62	0.94 (0.29–1.99)
C18:2 *n*-6 (*Linoleic Acid*, LA)	11.02	6.80	5.36	13.13	12.18	11.95	8.75	7.30	2.56	9.19	8.97 (2.56–13.13)
C20:4 *n*-6 (*Arachidonic Acid*, ARA)	0.26	0.10	0.02	0.40	0.26	0.18	0.05	0.07	0.23	0.17	0.18 (0.02–0.40)
Total LC-PUFAs *n*-6	11.32	6.97	5.45	13.59	12.56	12.19	8.83	7.41	2.92	9.40	9.12 (2.92–13.59)
Total PUFAs	12.58	7.82	5.98	14.65	13.10	14.34	9.72	8.45	3.25	11.06	10.39 (3.25–14.65)
Ratio LC-PUFAs *n*-6/LC-PUFAs *n*-3	9.3	8.4	10.7	13.5	25.1	6.1	10.1	7.2	10.1	5.8	9.7 (5.8–25.1)
***Trans* Fatty Acids (TFAs)** [% *wt*/*wt*]											
C16:1 *trans (trans-Palmitoleic Acid)*	0.20	0.16	0.14	0.39	0.26	0.21	0.09	0.09	0.18	0.11	0.17 (0.09–0.39)
C18:1 *trans (trans-Oleic Acid)*	0.27	0.96	1.09	0.46	0.76	0.40	0.40	0.69	1.47	0.33	0.58 (0.27–1.47)
C18:2 *trans (Linolelaidic Acid)*	0.05	0.20	0.31	0.14	0.13	0.04	0.17	0.18	0.46	0.06	0.16 (0.04–0.46)
Total TFAs	0.52	1.32	1.54	0.99	1.15	0.65	0.66	0.96	2.11	0.50	0.98 (0.50–2.11)

* Data for fatty acids are presented as the relative proportion of each fatty acid (% of total fatty acids—% *wt*/*wt*). ** LOQ (limit of quantification) for fatty acids = 0.01% *wt*/*wt*—½ LOQ was taken for the calculations, i.e., 0.005% *wt*/*wt*. Total SFAs—sum of saturated fatty acids (C4:0 + C6:0 + C8:0 + C10:0 + C11:0 + C12:0 + C14:0 + 15:0 + C16:0 + C17:0 + C18:0 + C20:0 + C22:0 + C24:0 + C21:0 + C23:0). Total MUFAs—sum of monounsaturated fatty acids (C14:1 + C15:1 + C16:1 cis + C17:1 + C18:1 cis + C20:1 + C22:1 + C24:1). Total PUFAs—sum of polyunsaturated fatty acids (C18:2 *n*-6 + C18:3 *n*-6 + C20:3 *n*-6 + C20:4 *n*-6 + C18:3 *n*-3 + C20:3 *n*-3 + C20:5 *n*-3 + C22:5 *n*-3 + C22:6 *n*-3+ C20:2 + C22:2). Total LC-PUFAs *n*-6—sum of long chain *n*-6 polyunsaturated fatty acids (C18:2 *n*-6 + C18:3 *n*-6 + C20:3 *n*-6 + C20:4 *n*-6). Total LC-PUFAs *n*-3—sum of long chain *n*-3 polyunsaturated fatty acids (C18:3 *n*-3 + C20:3 *n*-3 + C20:5 *n*-3 + C22:5 *n*-3 + C22:6 *n*-3). Total TFAs—sum of *trans* fatty acids (C16:1 *trans* + C18:1 *trans* + C18:2 *trans*).

**Table 3:** Comparison of nutrient content (g), dry matter (g) and energy value (kcal) of breast milk.

Parameter	Day 2	Day 3	Day 4	Day 5	Day 6	Day 7	Day 8	Day 9	Day 10	Day 11	Days 2–11 Median (Min–Max)
**Fat content in breast milk** (g/100 mL of breast milk)	4.0	5.6	6.4	6.0	4.5	4.4	5.4	4.4	3.1	5.3	4.9 (3.1–6.4)
**Total protein content in breast milk** (g/100 mL of breast milk)	4.1	5.2	5.1	4.5	3.7	5.4	5.5	5.0	2.0	4.6	4.8 (2.0–5.5)
**True protein content in breast milk** (g/100 mL of breast milk)	3.3	4.1	4.1	3.6	2.9	4.3	4.4	4.0	1.6	3.7	3.9 (1.6–4.4)
**Carbohydrate (lactose) content in breast milk** (g/100 mL of breast milk)	2.3	2.4	1.6	3.4	3.4	4.7	5.1	4.6	4.1	5.6	3.8 (1.6–5.6)
**Dry matter content in breast milk** (g/100 mL of breast milk)	10.7	13.3	13.3	14.1	11.8	14.7	16.2	14.3	9.4	15.7	13.7 (9.4–16.2)
**Energy value of breast milk** (kcal/100 mL of breast milk)	64	84	88	89	71	83	95	81	54	92	84 (54–95)

**Table 4:** Comparison of content of selected fatty acids, including TFAs (% *wt*/*wt*) ^1^ in breast milk samples.

Fatty Acids	Day 2	Day 3	Day 4	Day 5	Day 6	Day 7	Day 8	Day 9	Day 10	Day 11	Days 2–11 Median (Min–Max)
**Total Saturated Fatty Acids (SFAs)** [% *wt*/*wt*]											
C14:0 *(Myristic Acid)*	7.10	7.18	7.56	7.64	6.93	7.46	7.12	6.32	6.53	6.62	7.11 (6.32–7.64)
C16:0 *(Palmitic Acid)*	23.85	22.84	22.78	23.23	22.86	23.66	22.97	22.76	22.45	23.74	22.92 (22.45–23.85)
C18:0 *(Stearic Acid)*	7.68	8.13	8.79	8.72	8.45	8.62	7.99	7.55	7.03	7.65	8.06 (7.03–8.79)
Total SFAs	43.69	43.43	44.68	45.24	43.50	44.89	43.02	41.28	40.50	43.20	43.47 (40.50–45.24)
***cis*-Monounsaturated Fatty Acids (MUFAs)** [% *wt*/*wt*]											
C16:1 *cis (cis-Palmitoleic Acid)*	1.71	1.78	1.91	2.03	2.04	2.08	2.04	1.89	1.74	2.26	1.97 (1.71–2.26)
C18:1 *cis (cis-Oleic Acid)*	35.66	35.62	36.80	37.58	37.53	37.89	37.27	36.31	33.49	39.40	37.04 (33.49–39.40)
Total MUFAs	38.33	38.47	39.92	40.85	40.76	41.05	40.51	39.23	36.23	42.75	40.22 (36.23–42.75)
***cis*-Polyunsaturated Fatty Acids (PUFAs)** [% *wt*/*wt*]											
C18:3 *n*-3 (*α-Linolenic Acid*, ALA)	0.53	0.60	0.68	0.67	0.66	0.60	0.56	0.51	0.40	0.59	0.60 (0.40–0.68)
C20:5 *n*-3 (*Eicosapentaenoic Acid*, EPA)	0.03	0.04	0.05	0.05	0.06	0.06	0.05	0.04	0.04	0.04	0.05 (0.03–0.06)
C22:6 *n*-3 (*Docosahexaenoic Acid*, DHA)	0.83	0.64	0.61	0.68	0.62	0.79	0.90	0.85	1.12	0.79	0.79 (0.61–1.12)
Total LC-PUFAs *n*-3	1.44	1.35	1.44	1.81	1.87	2.20	2.37	2.25	2.53	1.91	1.89 (1.35–2.53)
C18:2 *n*-6 (*Linoleic Acid*, LA)	7.77	7.76	8.22	8.27	8.30	8.12	7.81	7.46	6.71	8.21	7.96 (6.71–8.30)
C20:4 *n*-6 (*Arachidonic Acid*, ARA)	0.29	0.37	0.41	0.41	0.42	0.41	0.39	0.36	0.31	0.37	0.38 (0.29–0.42)
Total LC-PUFAs *n*-6	8.22	8.30	8.84	8.89	8.93	8.70	8.35	7.95	7.12	8.72	8.53 (7.12–8.93)
Total PUFAs	9.76	9.81	10.47	10.89	10.99	11.06	10.88	10.33	9.74	10.79	10.63 (9.74–11.06)
Ratio LC-PUFAs *n*-6/LC-PUFAs *n*-3	5.7	6.1	6.1	4.9	4.8	4.0	3.5	3.5	2.8	4.6	4.7 (2.8–6.1)
***Trans* Fatty Acids (TFAs)** [% *wt*/*wt*]											
C16:1 *trans (trans-Palmitoleic Acid)*	0.29	0.33	0.39	0.36	0.36	0.36	0.33	0.30	0.25	0.33	0.33 (0.25–0.39)
C18:1 *trans (trans-Oleic Acid)*	0.77	0.50	0.60	0.60	0.55	0.51	0.40	0.36	0.26	0.37	0.51 (0.26–0.77)
C18:2 *trans (Linolelaidic Acid)*	0.06	0.08	0.14	0.15	0.14	0.14	0.09	0.08	0.07	0.10	0.10 (0.06–0.15)
Total TFAs	1.12	0.91	1.13	1.11	1.05	1.01	0.82	0.74	0.58	0.80	0.96 (0.58–1.13)

^1^ Data for fatty acids are presented as the relative proportion of each fatty acid (% of total fatty acids—% *wt*/*wt*). Total SFAs—sum of saturated fatty acids (C4:0 + C6:0 + C8:0 + C10:0 + C11:0 + C12:0 + C14:0 + 15:0 + C16:0 + C17:0 + C18:0 + C20:0 + C22:0 + C24:0 + C21:0 + C23:0). Total MUFAs—sum of monounsaturated fatty acids (C14:1 + C15:1 + C16:1 cis + C17:1 + C18:1 cis + C20:1 + C22:1 + C24:1). Total PUFAs—sum of polyunsaturated fatty acids (C18:2 *n*-6 + C18:3 *n*-6 + C20:3 *n*-6 + C20:4 *n*-6 + C18:3 *n*-3 + C20:3 *n*-3 + C20:5 *n*-3 + C22:5 *n*-3 + C22:6 *n*-3 + C20:2 + C22:2). Total LC-PUFAs *n*-6—sum of long chain *n*-6 polyunsaturated fatty acids (C18:2 *n*-6 + C18:3 *n*-6 + C20:3 *n*-6 + C20:4 *n*-6). Total LC-PUFAs *n*-3—sum of long chain *n*-3 polyunsaturated fatty acids (C18:3 *n*-3 + C20:3 *n*-3 + C20:5 *n*-3 + C22:5 *n*-3 + C22:6 *n*-3). Total TFAs—sum of *trans* fatty acids (C16:1 *trans* + C18:1 *trans* + C18:2 *trans*).

**Table 5:** Correlations between *trans* fatty acids content in the breastfeeding mother’s diet and *trans* fatty acids content in breast milk.

*Trans* Fatty Acids Content in Mother’s Diet	*Trans* Fatty Acids Content in Breast Milk				
	C16:1 *trans* (% *wt*/*wt*)	C18:1 *trans* (% *wt*/*wt*)	C18:2 *trans* (% *wt*/*wt*)	TFAs (% *wt*/*wt*)	TFAs (g/100 mL)
**C16:1 *trans*** (% *wt*/*wt*)	0.2699 *p = 0.451*	0.4725 *p = 0.168*	0.5526 *p = 0.098*	0.5335 *p = 0.112*	0.4972 *p = 0.144*
	0.3178 *p = 0.371*	0.5671 *p = 0.087*	0.4044 *p = 0.246*	0.4924 *p = 0.148*	0.4072 *p = 0.243*
**C16:1 *trans*** (g/reconstructed daily food ration)	0.2505 *p = 0.485*	0.7766 *p = 0.008*	0.3125 *p = 0.379*	0.7266 *p = 0.017*	0.5408 *p = 0.106*
	0.5281 *p = 0.117*	0.9179 *p = 0.001*	0.4801 *p = 0.160*	0.8545 *p = 0.002*	0.6608 *p = 0.038*
**C18:1 *trans*** (% *wt*/*wt*)	−0.2430 *p = 0.499*	−0.3975 *p = 0.255*	−0.1090 *p = 0.764*	−0.3878 *p = 0.268*	−0.1215 *p = 0.738*
	0.1122 *p = 0.758*	−0.2622 *p = 0.464*	0.0370 *p = 0.919*	−0.1398 *p = 0.700*	0.1263 *p = 0.728*
**C18:1 *trans*** (g/reconstructed daily food ration)	0.6801 *p = 0.030*	0.6032 *p = 0.065*	0.3701 *p = 0.292*	0.6938 *p = 0.026*	0.8420 *p = 0.002*
	0.5778 *p = 0.0802*	0.6991 *p = 0.025*	0.3447 *p = 0.329*	0.7576 *p = 0.011*	0.8748 *p = 0.001*
**C18:2 *trans*** (% *wt*/*wt*)	−0.3409 *p = 0.335*	−0.4687 *p = 0.172*	−0.1917 *p = 0.596*	−0.4806 *p = 0.160*	−0.1576 *p = 0.664*
	−0.1553 *p = 0.668*	−0.3951 *p = 0.258*	−0.2216 *p = 0.538*	−0.2848 *p = 0.425*	−0.0189 *p = 0.959*
**C18:2 *trans*** (g/reconstructed daily food ration)	0.4764 *p = 0.164*	0.2309 *p = 0.521*	0.2264 *p = 0.529*	0.3282 *p = 0.355*	0.6568 *p = 0.039*
	0.3293 *p = 0.353*	0.3465 *p = 0.327*	0.1846 *p = 0.610*	0.4182 *p = 0.229*	0.5790 *p = 0.079*
**TFAs** (% *wt*/*wt*)	−0.2237 *p = 0.534*	−0.3379 *p = 0.340*	−0.0332 *p = 0.928*	−0.3226 *p = 0.363*	−0.0440 *p = 0.904*
	0.1180 *p = 0.745*	−0.1094 *p = 0.763*	0.0369 *p = 0.919*	−0.0061 *p = 0.987*	0.2203 *p = 0.541*
**TFAs** (g/100 g reconstructed daily food ration)	0.4807 *p = 0.160*	0.2923 *p = 0.412*	0.5073 *p = 0.135*	0.4288 *p = 0.216*	0.4685 *p = 0.172*
	0.5770 *p = 0.081*	0.4032 *p = 0.248*	0.5307 *p = 0.114*	0.4623 *p = 0.179*	0.4175 *p = 0.230*
**TFAs** (g/reconstructed daily food ration)	0.6456 *p = 0.044*	0.7809 *p = 0.008*	0.4262 *p = 0.219*	0.8371 *p = 0.003*	0.9134 *p = 0.000*
	0.5654 *p = 0.089*	0.8875 *p = 0.001*	0.3631 *p = 0.302*	0.9152 *p = 0.000*	0.8560 *p = 0.002*

Data is presented as the Pearson correlation coefficient (r, first line) and Spearman’s rank correlation coefficient (r_S_, second line). A *p*-value below 0.05 was adopted as statistically significant. Notes: TFAs—*trans* fatty acids.

## Data Availability

The original contributions presented in this study are included in the article; further inquiries can be directed to the corresponding authors.

## Abbreviations

The following abbreviations are used in this manuscript:

TFAs	*trans* fatty acids
i-TFAs	industrially produced *trans* fatty acids
r-TFAs	ruminant *trans* fatty acids
SFAs	saturated fatty acids
MUFAs	monounsaturated fatty acids
PUFAs	polyunsaturated fatty acids
LC-PUFAs *n*-6	long chain *n*-6 polyunsaturated fatty acids
LC-PUFAs *n*-3	long chain *n*-3 polyunsaturated fatty acids
ALA	α-linolenic acid
EPA	eicosapentaenoic acid
DHA	docosahexaenoic acid
LA	linoleic acid
ARA	arachidonic acid
PHOs	partially hydrogenated oils
WHO	World Health Organization
EFSA	European Food Safety Authority
